# Differences in treatment for Alzheimer's disease between urban and rural areas in China

**DOI:** 10.3389/fneur.2022.996093

**Published:** 2022-09-29

**Authors:** Bei Li, Dejun Liu, Qiaoqin Wan, Can Sheng, Xiting Wang, Fangda Leng, Qing Peng, Ting Wang, Ailian Du, Feiqi Zhu, Dunzhu Mima, Huali Wang, Hengge Xie, Zhaoxia Wang, Haiqiang Jin, Yongan Sun

**Affiliations:** ^1^Department of Neurology, Peking University First Hospital, Peking University, Beijing, China; ^2^Health Division of Guard Bureau, General Office of CPC Central Committee, Beijing, China; ^3^School of Nursing, Peking University, Beijing, China; ^4^Department of Geriatrics, Peking University First Hospital, Beijing, China; ^5^Department of Neurology, Shanghai Tongren Hospital, Shanghai Jiao Tong University School of Medicine, Shanghai, China; ^6^Department of Neurology, Shenzhen Luohu People's Hospital, Shenzhen, China; ^7^Department of Neurology, People's Hospital of Tibet Autonomous Region, Lhasa, China; ^8^Department of Psychiatry, Peking University Sixth Hospital, Peking University, Beijing, China; ^9^Department of Neurology, Second Medical Center of Chinese PLA General Hospital, Beijing, China

**Keywords:** dementia, Alzheimer's, treatment, medication adherence, regional difference

## Abstract

**Introduction:**

In China, the increasing number of people with Alzheimer's disease (AD) poses a great challenge to families and the country. Economic and cultural differences cause a urban-rural gap in medical resources. This multicenter survey aimed to investigate the real-world practice of disease treatment among people with AD.

**Methods:**

People with AD and their caregivers from 30 provincial regions in mainland China were enrolled from October 2020 to December 2020 to be surveyed for their treatment experience. Logistic regression was used to explore the factors that influence medication adherence in all areas, urban areas, and rural areas.

**Results:**

In this survey, 1,427 participants came from urban areas, and 539 participants came from rural areas. Patients in urban areas were older (mean age 74 vs. 70, *p* = 0.001), less frequently had mild AD (36.0 vs. 52.1%, *p* < 0.001), and more often were cared for at professional institutions (8.8 vs. 3.2%, *p* < 0.001). In terms of pharmacotherapy, 77.8% of people accepted taking lifelong medication, whereas 61.3% of patients insisted on taking medications. Although 72.0% of rural people believed in taking lifelong medication, only 30.0% adhered to drug use. The major factors that influenced medication adherence for all patients with AD were regional distribution (*p* < 0.001, OR = 6.18, 95% CI: 4.93–7.74) and family earnings (*p* = 0.003, OR = 1.22, 95% CI: 1.07–1.38). In rural areas, family earnings (*p* = 0.008, OR = 1.44, 95% CI: 1.10–1.89) and severity of AD (*p* = 0.033, OR = 1.31, 95% CI: 1.02–1.68) were the main factors. Family earnings (*p* = 0.038, OR = 1.16, 95% CI: 1.01–1.34) was the only factor among urban areas. Among all non-pharmaceutical activities except for cognitive intervention, the participation rates of rural patients were significantly higher than those of urban patients (*p* < 0.05).

**Conclusion:**

Although national progress has been made in the public awareness of disease treatment, adequate diagnosis and medication adherence need to be prompted, especially in rural areas. Furthermore, lifelong treatment should be improved based on regional characteristics through the joint efforts of the government, health workers, and social volunteers.

## Introduction

Alzheimer's disease (AD), a major type of dementia, has become a great public health challenge (1). Globally, nearly 50 million people live with dementia (2). The results of one worldwide survey organized by the WHO showed that AD and other forms of dementia have become the 7th leading cause of death (3). In China, a surge in the elderly population has resulted in 9.83 million people aged 60 years or older suffering from AD (4). AD rose to the fifth leading cause of death in China (5). In the US, the annual total costs of AD were estimated to be $167.74 billion in 2015 and were predicted to reach $507.49 billion in 2030 and $1.89 trillion in 2050 (6). This projected growth of care and economic burden of AD arouses national attention to the management of AD.

In response to the Global Action Plan on Dementia 2017–2025 published by the WHO and ADI, the Chinese Government and dementia organizations have made great efforts in the development of diagnosis and treatment criteria, reinforcement of training dementia doctors, and cultivation of public awareness about this disease (7, 8). In 2020, the national guidelines for the diagnosis and treatment of mental disorders pointed to the therapeutic principles of AD, which included early diagnosis, timely treatment, and lifelong management (9). Regarding the treatment of AD, this guideline emphasized pharmaceutical and non-pharmaceutical therapy. Adherence to pharmacotherapy was the key to delaying the deterioration of cognitive ability. In addition, supplementary non-pharmacotherapy played a momentous role in alleviating behavioral and psychiatric symptoms. However, the urban-rural gap in the medical field in China presents challenges for national promotion. Compared to people living in urban areas, people in rural areas often have lower levels of income and education and more difficulties accessing advanced medicine and technology (7, 10). Patients with AD in urban and rural areas in China also have different experiences in the treatment of dementia (7). Thus, examining the real-world practice of disease treatment in different regions of China is essential to provide a basis to formulate regulations and minimize the regional gap.

In this study, we aimed to investigate the demographic and clinical characteristics as well as the treatment experience of patients with AD in urban and rural areas. According to the data of this national survey, various guidelines should be considered and adopted by policy-makers, health workers, and researchers in China and worldwide to improve patients' access and adherence to the treatment of AD.

## Method

### Study design and samples

This large-sample, multicenter, cross-sectional study surveyed participants from 30 provincial, municipal, and autonomous regions in mainland China between October 2020 and December 2020, including Zhejiang, Beijing, Shaanxi, Shanxi, Hunan, Hebei, Henan, Heilongjiang and other provinces. Patients who were diagnosed with probable AD dementia by cognitive experts from comprehensive psychiatric hospitals according to the criteria of the National Institute of Aging and Alzheimer's Association (NIA-AA) in 2011 were included (11). The tools of neuropsychological assessment for diagnosis of AD were based on the national guidelines for the diagnosis and treatment of mental disorders, such as MoCA, MMSE, ADAS-cog, CDR and ADL (9, 12). If patients had other neurological diseases that were related to cognitive impairment and major psychiatric disorders, they were excluded. After obtaining patients' or their caregivers' consent, they were enrolled in the completion of questionnaires.

### Measures and data collection

The demographic and clinical characteristics and treatment experiences of patients with AD were assessed in this study. The questionnaire was derived from the results of a previous survey (13, 14) and had been modified many times by several specialists according to the aim of this study.

The demographic and clinical characteristics of patients were assessed with a self-designed questionnaire, including gender, age, residential area, length of education, family earnings, medical insurance, severity of AD, duration since AD dementia diagnosis, and form of care. The urban and rural typologies for the Chinese geographical regions were defined based on the “Provisions on the Statistical Division of Urban and Rural Areas” produced by the National Bureau of Statistics, which considered the differences in demographic, social and economic development (15). This survey classified urban and rural areas according to the residential area of patients and the national standards for regional division. Family earnings referred to per capita monthly household income, which included salary, bonuses, and allowances. The severity of AD was estimated by neurologists based on patients' symptoms and neuroimaging data. The criterion of MTA, which ranks the degree of hippocampal atrophy, is considered a major indicator of disease severity (16). Unlike home care, where family members or paid caregivers played the primary caregiving roles, professional care was consisted of day care services provided by community health services, omnidirectional hosting offered by nursing homes, and respite care provided by professional caregivers.

The treatment experiences of patients with AD were assessed with a self-designed questionnaire. The status of treatment was assessed by several items, including attitude toward lifelong medication, behavior when taking AD medications, views on AD medications, and participation in non-pharmaceutical therapy. In addition, one item was augmented based on the pharmaceutical behavior of patients. If patients were currently taking medications, they completed the item about the duration of drug adherence. On the condition that patients had never started or withdrawn from pharmacotherapy, the participants would answer the questions about the reasons for their abstinence or withdrawal. AD medications in this study included cholinesterase inhibitors, memantine, and/or traditional Chinese medicines. Participation in non-pharmaceutical therapy was related to whether patients ever participated in adjunctive activities, such as physical activity, cognitive intervention, mental activity and musical therapy.

If the patients and their caregivers were willing to participate in this survey after the introduction of study purposes, they completed a questionnaire under the guidance of the diagnostic doctors who received comprehensive training. When patients were unable to cooperate, their caregivers were asked for assisting in completing questionnaires.

### Ethical consideration

This study was approved by the Ethics Committee of Peking University First Hospital. The right to informed consent, voluntary participation and confidentiality of responses were assured.

### Statistical analyses

First, all variables were subjected to descriptive analyses with the SPSS 23.0 software. For categorical variables, data are presented as the number of cases and percentages. Continuous variables are summarized as the means and standard deviations. Then, the *t*-sample test and chi-square test were used to calculate *p*-values for differences between urban and rural areas. Finally, binary logistic regression was used to explore the factors that influence medication adherence. Based on the pharmacotherapy behaviors, people who did not adhere to medication regimens were classified as never having taken or having withdrawn from taking medication. All the demographic and clinical variables that showed statistically significant difference in univariate analysis were filtered by backward method with likelihood ratio test.

## Results

### Demographic and clinical characteristics

Finally, a total of 2,813 questionnaires (1,674 from urban areas and 1,139 from rural areas) were collected, and 1,966 valid questionnaires (1,427 from urban areas and 539 from rural areas) remained for analysis. Questionnaires were eliminated for the incorrect and leaked filling of items. The demographic and clinical characteristics of the urban and rural patients are presented in Table 1.

**Table 1:** The demographic and clinical characteristics of the urban and rural people with AD.

**Variables**	**Urban areas** **(*n*, %)**	**Rural areas (*n*, %)**	p^*^	**Total (*n*, %)**
Overall	1,427 (100)	539 (100)		1,966 (100)
**Gender**			<0.001	
Male	556 (39.0)	278 (51.6)		834 (42.4)
Female	871 (61.0)	261 (48.4)		1,132 (57.6)
**Age, years**			<0.001	
40–59	115 (8.0)	94 (17.4)		209 (10.6)
60–79	827 (60.0)	326 (60.5)		1,153 (58.6)
≥80	485 (34.0)	119 (22.1)		604 (30.7)
**Length of education**			<0.001	
0–9 years	701 (49.1)	430 (79.8)		1,131 (57.5)
10–12 years	377 (26.4)	67 (12.4)		444 (22.6)
≥13 years	349 (24.5)	42 (7.8)		391(19.9)
**Family earnings (monthly)**			<0.001	
≤ 5,000 RMB	756 (53.0)	414 (76.8)		1,170 (59.5)
5,001–10,000 RMB	474 (33.2)	98 (18.2)		572 (29.1)
10,001–15,000 RMB	107 (7.5)	12 (2.2)		119 (6.1)
>15,000 RMB	90(6.3)	15 (2.8)		105( 5.3)
**Medical insurance**			<0.001	
Yes	803 (56.3)	176 (32.7)		979(49.8)
No	624 (43.7)	363 (67.3)		987 (50.2)
**Severity of AD**			<0.001	
Mild	514 (36.0)	281 (52.1)		795 (40.4)
Moderate	377 (26.4)	144 (26.7)		521 (26.5)
Severe	536 (37.6)	114 (21.2)		650 (33.1)
**Duration since AD dementia diagnosis**			<0.001	
<1 year	327 (22.9)	249 (46.2)		576 (29.3)
1–3 years	549 (38.5)	196 (36.4)		745 (37.9)
4–6 years	356 (24.9)	55 (10.2)		411 (20.9)
7–10 years	141 (9.9)	23 (4.3)		164 (8.3)
>10 years	54 (3.8)	16 (3.0)		70 (3.6)
**Form of care**			<0.001	
Care at home	1,301 (91.2)	522 (96.8)		1,823 (92.7)
Care from professional institutions	126 (8.8)	17 (3.2)		143 (7.3)

* p-values from Chi-square test.

Their average age was 73.1 ± 10.7 years. In addition, urban patients were, on average, older than rural patients (74.3 ± 10.0 vs. 70.0 ± 11.7; *t* = −8.132, *p* = 0.001). Moreover, 40.4% of patients had mild AD, whereas 33.1% of patients were classified as having severe AD. However, fewer people suffered from mild AD in urban areas than in rural areas (36.0 vs. 52.1%, *p* < 0.001). Among rural patients with low education (length of education: 0–9 years), 49.5% (*n* = 213) of them had mild AD, while 21.2% (*n* = 91) of them were sever. In urban areas, 35.0% (*n* = 245) of patients with low education were categorized as mild AD and 35.7% (*n* = 250) of them had sever AD. Moreover, the form of care significantly differed between urban and rural areas, and more patients obtained care from professional institutions in urban areas than in rural areas (8.8 vs. 3.2%, *p* < 0.001). A total of 92.7% of patients lived at home and were cared for by family members (*n* = 1,561, 1,072 from urban areas and 489 from rural areas) or paid caregivers (*n* = 262, 229 from urban areas and 33 from rural areas).

### Treatment of AD

The characteristics of the treatment experiences of the urban and rural patients are shown in Table 2.

**Table 2:** The characteristics about treatment of the urban and rural people with AD.

**Variables**	**Urban areas ** **(*n*, %)**	**Rural areas** **(*n*, %)**	p^*^	**Total** **(*n*, %)**
Attitude toward lifelong pharmacotherapy	1,427 (100)	539 (100)	<0.001	1,966 (100)
Agree	1,141 (80.0)	388 (72.0)		1,529 (77.8)
Disagree	286 (20.0)	151 (28.0)		437 (22.2)
Behavior when taking AD medications	1,427 (100)	539 (100)	<0.001	1,966 (100)
Medication adherence	1,044 (73.2)	162 (30.0)		1,206 (61.3)
Medication withdrawal	229 (16.0)	168 (31.2)		397 (20.2)
Never start	154 (10.8)	209 (38.8)		363 (18.5)
Duration of medication adherence	1,044 (100)	162 (100)	<0.001	1,206 (100)
<1 year	309 (29.6)	108 (66.7)		417 (34.6)
1–6 years	651 (62.4)	48 (29.6)		699 (58.0)
>6 year	84 (8.0)	6 (3.7)		90 (7.4)
Reasons for medication withdrawal	229 (100)	168 (100)		397 (100)
Poor curative effect	114 (49.8)	68 (40.5)	0.066	182 (45.8)
High price	53 (23.1)	68 (40.5)	<0.001	121 (30.5)
Inconvenience in taking medicine	34 (14.8)	57 (33.9)	<0.001	91 (22.9)
Adverse effects	64 (27.9)	20 (11.9)	<0.001	84 (21.2)
Afraid of adverse effects	45 (19.7)	37 (22.0)	0.564	82 (20.7)
Reluctance from patients	51 (22.3)	29 (17.3)	0.219	80 (20.2)
others	30 (13.1)	6 (3.6)	0.001	36 (9.1)
Reasons for not starting pharmacotherapy	154(100)	209(100)		363(100)
Mild of severity	46 (29.9)	89 (42.6)	0.013	135 (37.2)
Afraid of adverse effects	55 (35.7)	41 (19.6)	0.001	96 (26.4)
Poor curative effect	429 (27.3)	53 (25.4)	0.682	95 (26.2)
Without prescription	41 (26.6)	31 (14.8)	0.005	72 (19.8)
High price	259 (16.2)	44 (21.1)	0.247	69 (19.0)
Lack of medicine in local hospital	19 (12.3)	50 (23.9)	0.005	69 (19.0)
Reluctance from patients	12 (7.8)	24 (11.5)	0.245	36 (9.9)
Others	20 (13.0)	9 (4.3)	0.003	29 (8.0)
Views on AD medications	1,427 (100)	539 (100)		1,966 (100)
High price	971 (68.0)	310 (57.5)	<0.001	1,281 (65.2)
Poor curative effect	1,006 (70.5)	266 (49.4)	<0.001	1,272 (64.7)
Adverse effects	519 (36.4)	160 (29.7)	<0.001	679 (34.5)
Effects vary with the brands of drug	256 (17.9)	135 (25.0)	<0.001	391 (19.9)
Inconvenience in taking medicines	137 (9.6)	114 (21.2)	<0.001	251 (12.8)
Participation in non-pharmaceutical therapy	1,427 (100)	539 (100)		1,966 (100)
Regular physical activity	887 (62.2)	365 (67.7)	0.022	1,252 (63.7)
Cognitive intervention	812 (56.9)	331 (61.4)	0.071	1,143 (58.1)
Regular mental activity	645 (45.2)	304 (56.4)	<0.001	949 (48.3)
Musical therapy	587 (41.1)	270 (50.1)	<0.001	857 (43.6)
Traditional Chinese therapies	518 (36.3)	317 (58.8)	<0.001	835 (42.5)
Physical therapy	322 (22.6)	266 (49.4)	<0.001	588 (29.9)
Caelotherapy/ritual healing	280 (19.6)	207 (38.4)	<0.001	487 (24.8)
Operative treatment	154( 10.8)	151 (28.0)	<0.001	305 (15.5)

* p-values from Chi-square test.

In terms of pharmacotherapy, 77.8% of people agreed with lifelong medication, while 61.3% of patients insisted on taking medications. Moreover, 65.4% of people insisted on taking medications for more than 1 year. Their attitudes toward current medications were that the treatments were expensive (65.2%) and poorly effective (64.7%). In rural areas, 72.0% of people believed in lifelong medication, but only 30.0% of patients adhered to drug treatment. The most frequent reasons for medication withdrawal among rural people were high price (40.5%) and poor curative effect (40.5%). In addition, 42.6% of patients considered the severity of their cognitive impairment to be too mild to require medications. In urban areas, patients generally withdrew from or denied medication treatment because of a poor curative effect (49.8%) and a fear of adverse effects (35.7%). Moreover, the two most common non-pharmaceutical therapies were regular physical activity (urban areas: 62.2% vs. rural areas: 67.7%, *p* = 0.022) and cognitive intervention (urban areas: 56.9% vs. rural areas: 61.4%, *p* = 0.071). The participation rates of rural patients in all non-pharmaceutical activities except cognitive intervention were significantly higher than those of urban patients (*p* < 0.05).

### Factors that influence medication adherence

Table 3 shows the screening results of binary logistic regression models, which were built using the demographic and clinical characteristics from Table 1 and behavior of pharmacotherapy from Table 2 (people with medication adherence vs. people without medication adherence). The major factors that influence medication adherence for patients with AD were regional distribution (*p* < 0.001, OR = 6.18, 95% CI: 4.93–7.74) and family earnings (*p* = 0.003, OR = 1.22, 95% CI: 1.07–1.38). The results of further analyses with factors that influenced medication adherence in different areas showed that family earnings (*p* = 0.038, OR = 1.16, 95% CI: 1.01–1.34) was the only factor among urban areas, whereas family earnings (*p* = 0.008, OR = 1.44, 95% CI: 1.10–1.89) and the severity of AD (*p* = 0.033, OR = 1.31, 95% CI: 1.02–1.68) were the main factors for rural people.

**Table 3:** Binary logistic regressions of medication adherence among the urban and rural people with AD.

**Variables in different models**	***p***	**OR (95% CI)**	***X* ^2^**	***p***
Model in overall areas			6.672	<0.001
Areas	<0.001	6.18(4.93–7.74)		
Age, years	0.076	0.86(0.73–1.02)		
Family earning (monthly)	0.003	1.22(1.07–1.38)		
Constant	<0.001	0.07		
Model in urban areas			5.418	0.035
Family earning (monthly)	0.038	1.16(1.01–1.34)		
Constant	<0.001	2.13		
Model in rural areas			6.525	0.001
Family earning (monthly)	0.008	1.44(1.10–1.89)		
Severity of AD	0.033	1.31(1.02–1.68)		
Duration since AD dementia diagnosis	0.052	0.81(0.65–1.00)		
Form of care	0.062	0.14(0.02–1.10)		
Constant	<0.001	0.25		

In all analyses, people without medication adherence is the reference category.

## Discussion

The increasing incidence of AD among the aging population is imposing a significant public health burden in China. The national government and related organizations have made great efforts in the development of practice guidelines, professional training and public education in the last decade (4, 7). However, this study is, to the authors' best knowledge, the first national cross-sectional survey that explored the characteristics and influencing factors of treatment among people with AD from urban and rural areas in China. The results identified differences in the pharmacological and non-pharmacological therapies employed between urban and rural areas, especially in terms of medication compliance.

In our survey, more than 70% of people agreed with lifelong medication, irrespective of their origin (rural or urban areas). Over 80% of all patients had used at least one antidementia drug. The treatment rate was appreciably higher than that reported by a previous study conducted in China 10 years ago, which showed that 30.1% of people with AD were treated with medication (17). This achievement would not be possible without mutual efforts by the government, hospitals, institutions and media to raise public awareness (18). In addition, ~60% of patients in our survey insisted on drug treatment, 65% of whom had been taking medications for more than 1 year. However, only 30% of rural patients in our research adhered to a medication regimen, and more than 60% of patients consistently used medication for less than a year. A previous survey indicated that the rates of medication compliance ranged from 17 to 100% among people with AD, which might be attributed to cognitive deficits in various domains, unique sociocultural backgrounds, and different reporters (19–21). Given that the prompt initiation of and adherence to medication regimens after diagnosis are crucial to cognitive preservation and achieving maximal treatment efficacy (9, 22), pharmacotherapy compliance needs to be further improved in China, especially in rural areas, as it is determined by specific individual characteristics.

The logistic results of this study found that regional factors and family earnings primarily influenced medication adherence. The survey also showed regional differences in the reasons for non-compliance with medication regimens. In terms of reasons for not starting pharmacotherapy, nearly half of rural participants thought their illness was not sufficiently serious to require medication, while most urban participants were afraid of the adverse effects. Unlike in urban areas, 40% of patients in rural areas cannot afford to adhere to a medication regimen. Imbalances in economic and cultural development in different regions of China might cause discrepancies in access to medical resources (10). Conversely, a shortage of dementia doctors and memory clinics in rural areas could prevent some patients with mild AD from accessing care, resulting in a low number of prescriptions for treating AD (7, 17). Consequently, patients did not realize the importance of taking medication. Similar to the results of other studies, awareness of the disease and an emphasis on medication were positively related to treatment adherence (23, 24). Moreover, challenges in obtaining financial reimbursement for drug costs may have influenced access to AD treatment, which also corroborated previous studies (19, 25). Hence, financial support, professional training, the implementation of memory clinics and popularization of knowledge about disease among rural areas may alleviate regional gaps in pharmacotherapy adherence.

Because most rural participants were younger and at an earlier stage of AD than urban participants, pharmacotherapy might be more effective for this population in preventing cognitive decline and maintaining the ability to complete daily activities (9). The logistic analysis of this study concentrating on rural patients illustrated that family earnings and the severity of AD influenced medication adherence. Economic factors for non-compliance to AD medications were coincident with the classification by the WHO (19, 26). In addition, AD with memory loss and impaired executive function was closely related to medication adherence (21). A previous study explored whether the differential outcome procedure that attempts to improve discriminative learning and memory might simulate adherence to medical prescriptions (27). Because of these symptoms of AD, social support from caregivers is important to ensure adherence to a medication regimen. Family members, especially spouses, can supervise patients' medications and even expand their role to provide emotional support and manage disruptive behaviors (23, 28). Overall, not only financial support but also cognitive training programs and caregiver support should be improved to increase medication adherence in rural areas.

In accordance with rural people, family earnings was a factor that influenced medication adherence for urban people. But apart from that, this study identified reasons for non-compliance among these patients that could not be neglected. Most urban participants did not start pharmacotherapy due to a fear of adverse effects and suspended AD medications because of poor curative effects. In accordance with other studies, side effects from medications, including diarrhea, nausea, sleep disturbances and ineffectiveness, contributed to non-compliance (9, 19, 29). Older people with comorbidities usually took multiple medications and consequently worried about health outcomes, such as longer survival, prevention of disease-specific events, and tolerable risk of adverse drug reactions. Therefore, they preferentially adhered to treatment for chronic deases other than AD, such as hypertension, diabetes mellitus and dyslipidemia (19, 21). Moreover, negative therapeutic experiences due to adverse drug effects and the ineffectiveness of medication might be due to the mode of use and inadequate pharmaceutical form for the clinical condition, such as dysphagia (29, 30). Combination medication with augmented therapeutic benefits and replacement with transdermal rivastigmine might be useful to ease the worries of patients and their caregivers (31, 32). Therefore, appropriate medication guidance by a multidisciplinary team and a suitable medication form might be useful to facilitate adherence to a drug regimen among urban patients.

In addition to pharmacotherapy, non-pharmacological therapy is essential to postpone cognitive decline and manage neuropsychiatric symptoms. In our survey, more than half of respondents participated in non-pharmacological activities, such as cognitive intervention and regular physical activity. Cognitive training and exercise were recognized as common interventions once AD was diagnosed, despite a lack of evidence for the optimal mode of these activities to maximize benefit (22). Nevertheless, the findings indicated that a majority of interventions involved fewer urban patients than rural patients, which might be attributed to the characteristics of urban patients, who were older and had more serious AD characterized by impaired execution of daily activities and neuropsychiatric symptoms. Furthermore, the heavy traffic in urban areas hinders patients from leaving their homes to participate in various activities. Caregivers in previous Chinese studies also expressed the urgent need for formal support for caregiving (14, 33). In brief, the primary health service requires further improvement in terms of professional advice and convenient access to non-pharmacological therapy to ease the burden on caregivers.

The present study had several limitations. First, this study adopted a convenience sampling method, and 27.4% of participants came from rural areas, which might restrict its generalization in rural areas; however, this result may suggest that AD is underdiagnosed in rural areas. Second, this study was based on patient self-reports and interviews with their caregivers, and recall bias may have influenced the reported experiences of treatment. Third, this study defined rural or urban areas according to resident population, which might restrict direct comparisons with studies from other countries that rely on different definitions of living areas. Fourth, except for neuroimaging examination, patients with various demographic and clinical characteristics were assessed by different neuropsychological tools. In order to enhance professional training with diagnosis and management of dementia, future research could develop a national uniform system of neuropsychological evaluation for AD or other types of dementia. Finally, this survey adopted self-designed questionnaires which originated from the results of a previous survey and were modified by several specialists. Even though the results had astriction in comparability with studies from other countries, the characteristics of treatment among people with AD from urban and rural areas in China were revealed. Furthermore, people with mild cognitive impairment (MCI) were not included in this survey. People with MCI were encouraged to improve healthy lifestyle and prevent the occurrence of dementia (22). The management status of MCI could be investigated in the future. There is a window of intervention for delaying the progression of cognitive impairment.

## Conclusion

This study is the first to feature a large sample to examine differences in the characteristics of disease treatment in patients with AD in rural and urban areas and reflects the epidemiological reality in China. To achieve the Healthy China Initiative (2019–2030) (8), and there is still a pressing need to promote adequate diagnosis and treatment, especially in rural areas. Furthermore, lifelong treatment, including pharmacotherapy and non-pharmacological therapy, should be improved based on the characteristics of urban and rural areas through the joint efforts of government, health workers, and social volunteers.

## Data availability statement

The raw data supporting the conclusions of this article will be made available by the authors, without undue reservation.

## Ethics statement

The studies involving human participants were reviewed and approved by Ethics Committee of Peking University First Hospital. Written informed consent for participation was not required for this study in accordance with the national legislation and the institutional requirements.

## Author contributions

BL and DL contributed to the analysis and interpretation of the data and drafting and revision of the manuscript. CS, XW, FL, QP, TW, AD, FZ, and DM contributed to collection of data, quality control, and establishing the database. QW, HW, and HX contributed to advertising and coordination of the study. ZW, HJ, and YS contributed to the conceptualization of the study, formulation of study protocol, and intellectual revision of the manuscript. All authors contributed to the article and approved the submitted version.

## Funding

The study was supported by Science and Technology Innovation 2030- Major Project (2021ZD0201800, 2021ZD0201805).

## Conflict of interest

The authors declare that the research was conducted in the absence of any commercial or financial relationships that could be construed as a potential conflict of interest.

## Publisher's note

All claims expressed in this article are solely those of the authors and do not necessarily represent those of their affiliated organizations, or those of the publisher, the editors and the reviewers. Any product that may be evaluated in this article, or claim that may be made by its manufacturer, is not guaranteed or endorsed by the publisher.
